# New Appliances and Things Medical

**Published:** 1910-11-05

**Authors:** 


### NEW APPLIANCES AND THINGS MEDICAL.

[We shall be glad to receive at our Office, 28 & 29 Southampton Street, Strand, London, W.C., from the manufacturers, specimens of all new preparations and appliances.]

### OATMEAL STOUT.

(MESSRS. A. C. S. AND H. CROWLEY, Croydon.)

THE samples we have received of Crowley's Oatmeal Stout are excellent examples of this beverage both in quality and flavour. It is claimed that the employment of oats in combination adds greatly to its nutritive value, and there is no doubt that it would be of advantage in cases where such an aid to nutrition is indicated. The stout is guaranteed to keep indefinitely in any climate and to be absolutely pure and free from preservatives. The samples before us are certainly of high quality, and under the direction of the medical man should prove of service.

### THE FARRINGDON OVAL POCKET SALIVA-BOTTLE.

(H. POTHS & Co., 26 & 27 Farringdon Street, E.C.)

WE are indebted to the makers for kindly sending us a specimen of this exceedingly useful pocket spitting-bottle. The objectionable and even dangerous habit of promiscuous spitting is, thanks to the energetic crusade which has been waged against it, now on the decrease. We must always remember that, however dangerous the spitting of infectious patients is to the community, the swallowing of their own expectoration is in many cases most harmful to the individuals. Secondary infection of adjacent organs and chronic gastric and intestinal trouble are by no means infrequent results of this pernicious habit. Under the climatic conditions obtaining in this country catarrhal lesions of the respiratory tract, with the consequent necessity for coughing and expectorating, are very frequent. Hence we regard a small, cheap, and easily cleansable pocket spitting-flask, such as the one we have before us, as most desirable. It should certainly be carried by all those suffering from respiratory catarrh, whether of a tubercular nature or not.

### INSTITUTIONAL MACHINERY.

(MANLOVE, ALLIOTT AND Co., LTD., NOTTINGHAM.)

THE catalogue issued by the above firm is one that demands the consideration of institutional architects and those who have had anything to do with the furnishing of machinery rooms and the providing of special plant and apparatus for hospitals. It gives illustrations and details of various kinds of plant, ranging from accumulators, armature dryers, disinfectors, and cooking apparatus, to sewage plant, vacuum plant, and refuse destructors. Specimens of the apparatus supplied by the firm may be seen at many of our first-class hospitals and public institutions, and where they have been fitted they have always given satisfaction. A large number of varieties of each special class of plant are stocked, while modifications are frequently made to order. For hard wear and durability it would be difficult to beat the machinery turned out by this well-known Nottingham house, whose circular may be cordially recommended to those interested in such things. The London office of the firm is 42 and 43 Parliament Street, S.W.

